# The effects of regenerative injection therapy compared to corticosteroids for the treatment of lateral Epicondylitis: a systematic review and meta-analysis

**DOI:** 10.1186/s40945-019-0063-6

**Published:** 2019-11-13

**Authors:** Julie Barnett, Madison N. Bernacki, Jessica L. Kainer, Hannah N. Smith, Annette M. Zaharoff, Sandeep K. Subramanian

**Affiliations:** 1 Department of Physical Therapy, School of Health Professions, UT Health San Antonio, 7703 Floyd Curl Drive, MSC 6247, San Antonio, TX 78229 USA; 2 The Non-Surgical Center of Texas, San Antonio, USA

**Keywords:** Platelet rich plasma, Autologous blood injection, Elbow, Tendinosis, Enthesopathy

## Abstract

**Background:**

The lateral epicondyle is a common site for chronic tendinosis (i.e. lateral epicondylitis), a condition characterized by overuse and degeneration of a tendon due to repeated microtrauma. This leads to pain and functional limitations. There is a growing interest in non-surgical forms of treatment for this condition including provision of corticosteroid injections and regenerative injection therapy (provision of autologous blood and platelet rich plasma injections).

**Objective:**

We compared the effectiveness of corticosteroids with regenerative injection therapy for the treatment of lateral epicondylitis.

**Methods:**

We systematically reviewed randomized controlled trials published in English language from 2008 to 2018. Databases used included PEDro, Scopus, PubMed, and CINAHL. Nine articles met our selection criteria. The PEDRo scale scores helped assess study quality. Cochrane risk of bias criteria helped assess bias. We analyzed results focusing on pain and function using meta-analyses.

**Results:**

Six out of 9 studies had low risk of bias. There were no short-term (1 and 2 month) differences in pain scores between the corticosteroid and regenerative injection groups. Participants receiving regenerative injections demonstrated greater long-term improvements lasting for a period of **≈2** years.

**Conclusion:**

Regenerative injections provision results in greater long–term pain relief and improved function for people with lateral epicondylitis.

## Introduction

Lateral epicondylitis or tennis elbow is a relatively common condition for which people seek treatment. It is a disorder, which severely affects an individual’s function and mobility and results in multiple visits to orthopedic clinicians each year. This condition has an estimated incidence of 15.1 cases per 10,000 patients seen [1]. This condition is caused by repetitive microtrauma resulting in tendon degeneration [2]. Overexertion of the extremity with repetitive movements of wrist extension and alternating forearm pronation/supination are the main contributing factors to the repetitive microtrauma [3]. Traditionally, the condition is managed conservatively [4], with only a small proportion of individuals seeking surgery to alleviate pain [5]. Conservative treatment options including rest, bracing, prescription of non-steroidal anti-inflammatory drugs [6], provision of physiotherapy [7] and extracorporeal shock wave therapy [8]. Other conservative management options include the use of corticosteroid injections and regenerative injections. The injections are utilized as a means to postpone and/or prevent surgical intervention.

### Corticosteroid injections

One common conservative non-surgical option for the management of lateral epicondylitis involves the use of corticosteroid injections [9]. Corticosteroid injections work by down-regulation of immune function and reduction of inflammatory cells and mediators, such as lymphocytes, macrophages and mast cells [10]. Essentially, corticosteroid injections reduce pain caused by inflammation and are delivered using intra-articular or extra-articular injections. While intra-articular injections are used for conditions such as osteoarthritis, extra-articular (soft tissue) injections are used to target areas outside the joint and can be useful for tendinosis when they are injected directly into or in the area around the tendon [11]. However, corticosteroid injections increase protein catabolism, decrease type I collagen and glycosaminoglycan syntheses, and therefore slow the healing process [10]. Taking into consideration the lack of inflammation in cases of tendinosis and inhibition of collagen repair by corticosteroids, the utility of these injections for long-term symptom resolution in chronic tendinosis has been questioned at locations such as the Achilles tendon [12].

### Regenerative injections

The use of regenerative injections has been emerging as a conservative treatment method to not only treat musculoskeletal injuries, but also facilitate tissue regeneration and healing [13]. Common forms of regenerative injections include autologous blood injections or autologous conditioned plasma, platelet rich plasma, and prolotherapy. This treatment is based on the theory of introducing blood and platelet products to initiate maturation and proliferation within the tendon [14]. The regenerative injections stimulate cellular activity to initiate a healing response by increasing growth factors by increasing platelets or aseptic inflammation to trigger the reparative process of tendons [15]. It is possible to deliver the injections using ultrasound guidance to target either the most affected portion of the tendon or the point of tenderness. Each injection is prepared differently and is discussed below briefly.

### Autologous blood injections

Autologous blood injections involve performing a peripheral blood draw to extract blood from a distal site and injecting it directly into the affected tendon. A prospective observational cohort study by Edwards and Candruccio [15] evaluated the effects of autologous blood injections in the treatment of refractory lateral epicondylitis. The authors concluded autologous blood is an alternative minimally invasive treatment that is beneficial for patients with lateral epicondylitis with other failed conservative options.

### Platelet rich plasma injections

Platelet rich plasma is a biological blood product derived from centrifuged whole blood to extract concentrated platelets. The platelets stimulate the release of many growth factors imperative for tissue recovery and regeneration. They are injected directly into the site of injury to promote tissue healing [16]. Platelet rich plasma consists of concentrated platelets 5-10x the baseline amount in normal blood, which allows for increased tendon repair at the localized site of injection. Compared to surgical interventions that cost around $16,000 [4], the cost of platelet rich plasma injections range from $800–1200 per injection with 1–2 injections providing significant relief of pain and improvement in physiologic tissue recovery [17]. However, there is limited evidence currently available regarding standardized protocols and efficacy of platelet rich plasma with specific injuries [18]. However, platelet rich plasma may be a beneficial conservative treatment option for those seeking to postpone or prevent a surgical procedure.

### Surgery

About 10% of lateral epicondylitis patients will require some sort of surgical management because of inadequate relief from non-surgical measures [19]. Surgery is an option that many patients choose to delay in favor of conservative treatment. This decision can minimize personal financial hardship, decrease fear, and prevent adverse effects. Surgery involves removal of unhealthy tissue and promotes tendon healing [20]. Common procedures include open surgical debridement as well as arthroscopic tendon repairs. Reviews of surgical procedures for lateral epicondylitis have reported mixed evidence on the benefit of surgical interventions for lateral epicondylitis [19, 21]. The primary reason for the mixed evidence was the lack of well-controlled studies using direct comparisons between surgical procedures.

Given that a large proportion of individuals with lateral epicondylitis do not require surgical management, the purpose of this systematic review and meta-analysis is to investigate the effectiveness of two commonly used non-surgical options, regenerative injections and corticosteroid injections in the management of lateral epicondylitis. The question guiding our review in the Participant, Intervention, Comparison and Outcome (PICO) format [22] was “*In participants with lateral epicondylitis, does using regenerative injections result in lower pain and better functional outcomes compared to corticosteroid injections?*” Preliminary results have appeared in the abstract form [23].

## Materials and methods

A comprehensive literature review was conducted using the databases PubMed, PEDro, Scopus, and Ovid with time parameters from June 2008 to May 2018. The following search terms were utilized in varying combinations: ‘chronic tendinopathy’, ‘regenerative injection therapy’, corticosteroid injection’, ‘platelet rich plasma’, and ‘autologous blood injections’. The search was limited to availability of full text, human subjects, and English language. Inclusion criteria were: i) study design of randomized control trials (RCTs), ii) the comparison of regenerative injections with corticosteroid injections for lateral epicondylitis, and iii) the outcomes of pain and function. We excluded articles focusing on use of corticosteroids and/or regenerative injections for other joint conditions (e.g. knee OA). We reviewed all abstracts to select relevant articles and assessed the bibliography of each study to find any other relevant articles.

Each article was read independently by two of three (MNB, JLK and HNS) authors to determine if all inclusion criteria were met. In case of any discrepancy, a third author helped make the decision. We performed data extraction to obtain study design, population, intervention, outcome measure, results, and limitations. The quality of the published studies was evaluated using PEDro scale [24]. The scoring was completed by two of three authors, as mentioned before and discrepancies, if any, resolved by JBB and SKS. We interpreted the quality of the RCTs based upon the assessment of Foley et al. [25], where scores to 9–10, 6–8, 4–5 and ≤ 3/10 reflected *‘excellent’*, *‘good’*, *‘fair’* and *‘poor’* study quality respectively. Although utilized primarily in trials reporting neurorehabilitation interventions, use of the quality ratings based on PEDro scores is slowly increasing in the musculoskeletal rehabilitation literature [26–28]. We also performed a risk of bias assessment of included studies using the Cochrane Risk of Bias criteria for Effective Practice and Organization of Care reviews [29, 30]. Domains assessed included sequence generation, allocation concealment, blinding of participants, personnel and outcome assessors, incomplete outcome data, selective outcome reporting, and other sources of bias. For each domain, we assigned a judgment: *Yes -* indicating low risk of bias, *No -* indicating a high risk of bias, and *Unclear -* indicating unclear or unknown risk of bias where reported details were insufficient to reach a conclusion.

We calculated descriptive statistics of the study populations in terms of age, sex and time since diagnosis. All studies used outcomes at two International Classification of Functioning (ICF) levels [31] including impairment and limitations in activity performance. The underlying measurement construct for all outcomes was continuous. Meta-analyses (RevMan 5.3, Review Manager, Cochrane Collaboration, London, UK) examined the change in clinical outcomes after the provision of corticosteroids and regenerative injections. Outcomes included in the meta-analyses comprised of those used to measure change in two or more studies (the minimum number to be included in a meta-analysis) [32]. In studies using multiple outcomes, we conducted separate meta-analyses for each of the outcomes. Standardized mean differences (using Hedges g) and 95% confidence intervals helped quantify the pooled effects of the interventions. We calculated effect sizes to help quantify intervention effectiveness [33]. We assessed heterogeneity using tau-squared values (within study variance) and I^2^ (the ratio of true heterogeneity to total observed variation) values [34]. Studies were deemed heterogeneous if I^2^ values exceeded 50% [35]. Given the use of both autologous blood and protein rich plasma injections; we used the random effects model.

## Results

**Fig. 1:**
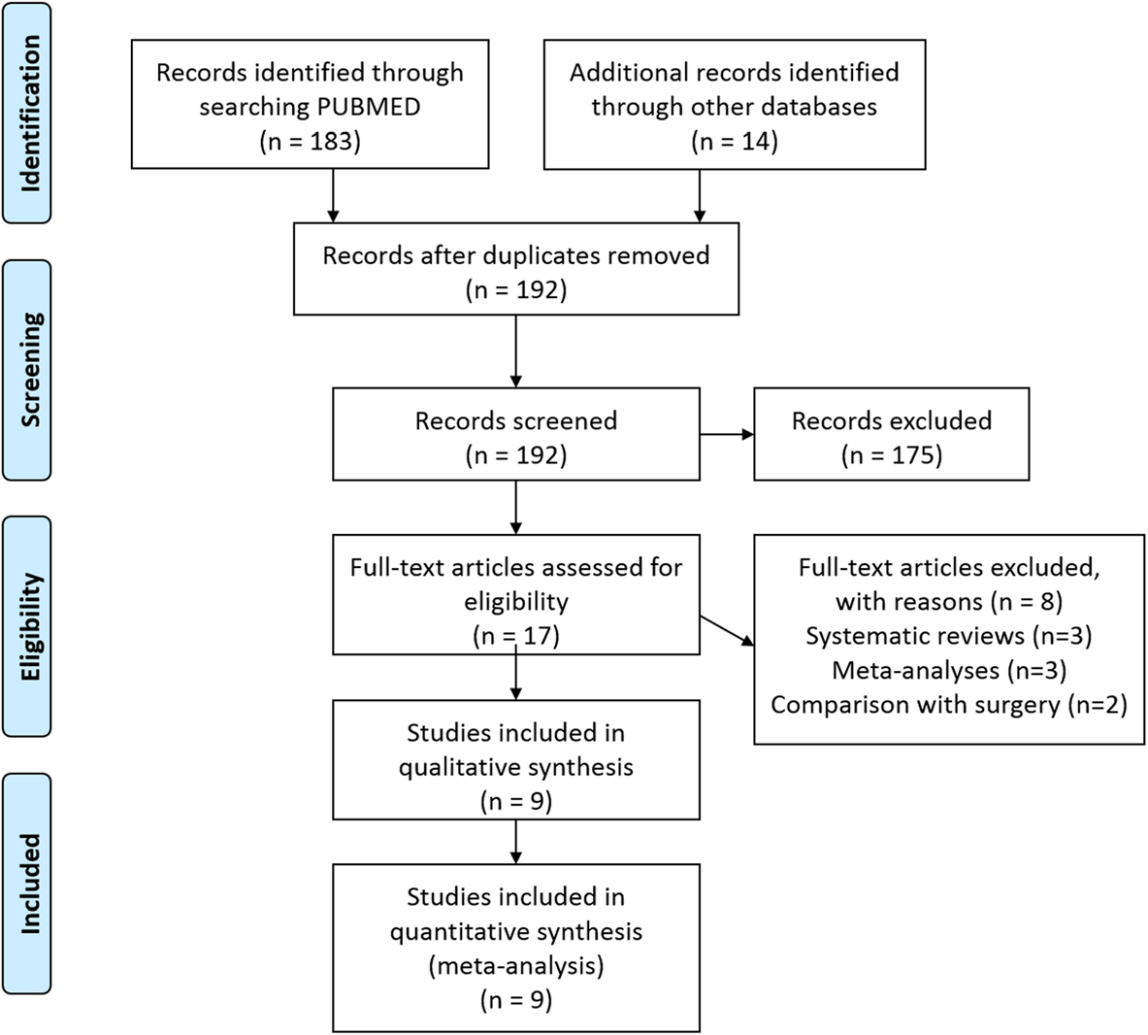
PRISMA Flow Diagram

The Preferred Reporting Items for Systematic Reviews and Meta-Analyses (PRISMA) flow diagram provides details of the search process and the selection results (Fig. 1). After initial screening, we selected seventeen articles. Eight articles were excluded, as they did not meet the inclusion criteria. The remaining nine articles were suitable for the study. The reference lists of these nine articles did not yield any additional citations. All nine of these studies were included in both the meta-analysis and qualitative (systematic review part) of the study.

### Main study characteristics

Five hundred and seventy-seven individuals with lateral epicondylitis participated in the nine studies included in the analysis. All studies included participants diagnosed with lateral epicondylitis at a mean of ≥ 2 months. The participants were predominantly female (60%) and the age range was from 36 to 54 years.

### Study quality, evidence levels and risk of Bias assessment

**Fig. 2:**
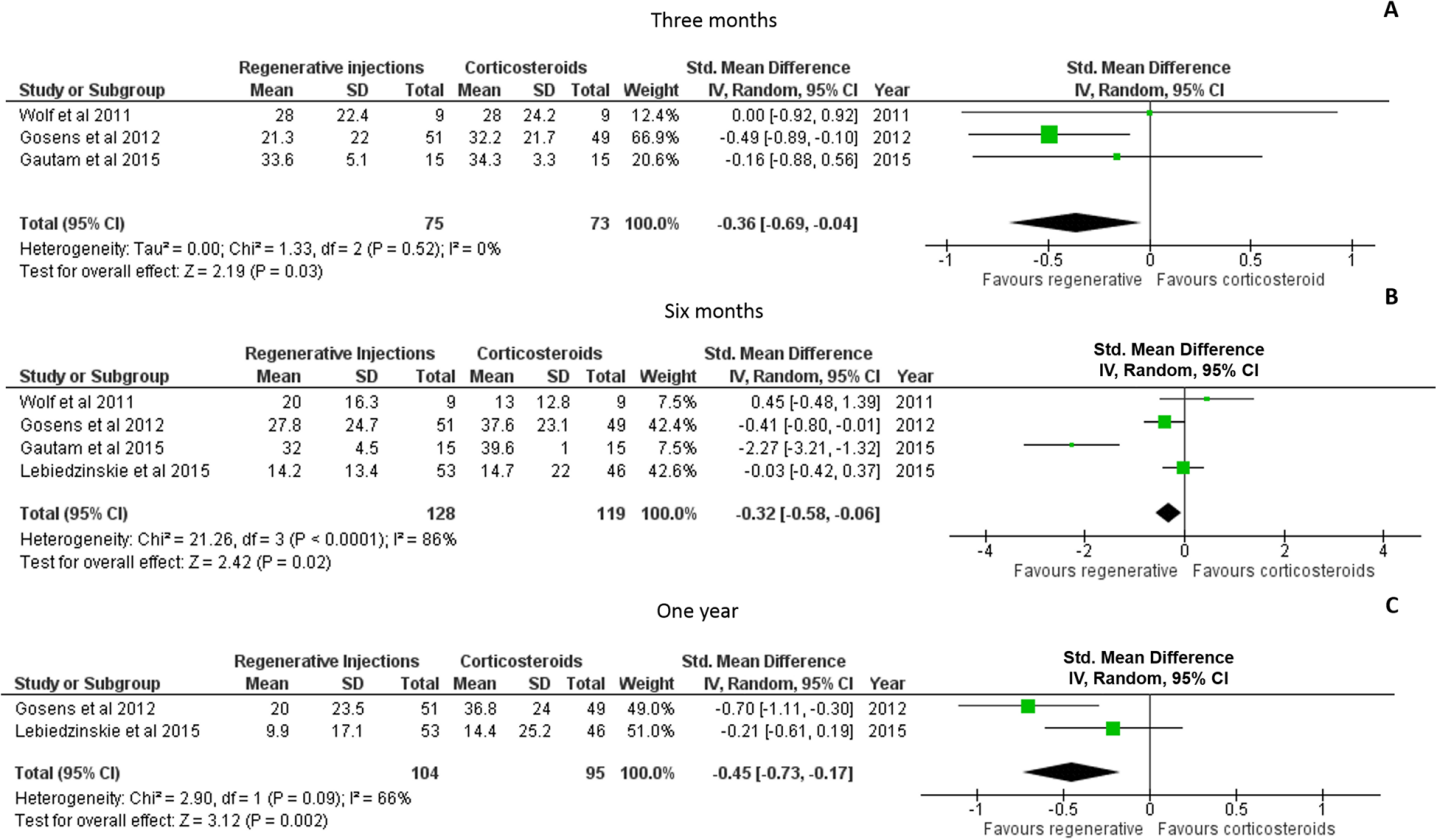
Risk of bias summary for included RCTs

The study quality was *excellent* (one study; PEDro score = 9) [36], *good* (six studies; PEDRo score = 6 for five studies and 8 for one study), [37–42] or *fair* (two studies; PEDro = 5) [43, 44] according to PEDro scale scores. There is evidence that provision of regenerative injections in the form of both autologous blood (three *good* quality studies; PEDro score 6/10) [37–39] and platelet rich plasma (one *excellent* study; PEDro score 9/10 [36]; and one *good* study; PEDro score 6/10 [41] decreases pain and improves function). Study details in terms of the numbers of participants, ages, the interventions (including type of injections received and site), outcomes, results and conclusions are available in Tables 1 and 2. Table 1 lists studies comparing autologous blood compared to corticosteroid injections and Table 2 list the studies comparing platelet rich plasma to corticosteroid injections. Figure 2 represents the summary information on the risk of bias assessment. All studies had a low risk for attrition bias and reporting bias and all but one for other bias. In terms of selection bias, information on random sequence allocation was missing in three studies, [41, 43, 44]. In addition, information on allocation concealment was unclear in two studies [39, 43], which potentially gives rise to a risk of high selection bias, particularly for the study by Arik and colleagues [43]. Information on performance bias in terms of blinding of personnel was unclear in five studies [36–39, 43]. Similarly, information on detection bias (blinding of outcome assessment) was missing in five studies [38, 39, 42–44]. This can also be a matter of concern, as not having blinded assessors can tend to influence the eventual results of the study. In terms of other bias, information on age and sex distribution was missing in one study [41].

**Table 1:** Details of studies comparing autologous blood and corticosteroid injections

Reference/ PEDro score Sackett’s Evidence Level	Participants	Methods	Outcome Measures	Results
Kazemi et al. (2010), PEDro = 6,	*n* = 60 participants; AB group: 30; Age: 47.2 ± 10.6 yrs. 7 males, 23 females CS group: 30 Age: 47.0 ± 10.3 yrs. 4 males, 26 females	• Participants received one injection into the lateral epicondyle region just inferior to the ECRB. • Two ml of AB mixed with one ml of 2% lidocaine injected in the AB group, while 20 mg methylprednisolone with one ml of 2% lidocaine provided in CS group. • Outcomes assessed before and after injection (four- and 8-weeks post-injection).	• VAS • Pain and strength in maximum grip • Quick DASH scores • Modified Nirschl scores • Pressure pain threshold	• Limb pain at rest, Quick DASH scores and pain in maximum grip lower in AB group at 4 weeks. (*p* < 0.01) • All the outcome measures significantly better in the AB group at 8 weeks evaluation (*p* < 0.001).
Dojode 2012 PEDro = 6	*n* = 60 participants AB group: 30 Mean Age: 42.9 yrs. 13 males, 17 females CS group: 30 Mean Age: 42.2 yrs. 12 males, 18 females	• All participants received injections at the lateral epicondyle into the undersurface of the ECRB. • AB: two ml drawn from the contralateral upper limb vein mixed with one ml 0.5% bupivacaine. • CS: two ml of methyl prednisolone acetate (80 mg) mixed with one ml 0.5% bupivacaine, at the lateral epicondyle. • Outcomes assessed pre-injection and at one, 4, 12 weeks and 6 months following the injection.	• VAS, • Nirschl staging of lateral epicondylitis	• CS group showed statistically significant decrease in pain compared to AB group in both outcomes at one and 4 weeks. • At 12 weeks, the VAS and Nirschl scores were significantly lower in the AB group (*p* = 0.0127 and *p* = 0.0184, respectively); this was maintained at 6 months • At final retention assessment, 47% in CS group and 90% in AB group were completely relieved of pain.
Arik et al. (2014) PEDro = 5	*n* = 80 participants AB group: 40; Age: 43.7 ± 7.8 yrs. 11 males, 29 females CS group: 40 Age: 46.7 ± 8.4 yrs. 10 males, 30 females	• Participants were given a single injection of AB (two ml venous blood collected from antecubital fossa of ipsilateral side mixed with one ml of 2% prilocaine hydrochloride) or CS injections (one ml of 40 mg methylprednisolone acetate mixed with one ml of 2% prilocaine hydrochloride). • Each participant was assessed before treatment and at day 15, 30, and 90 after the injection.	• VAS • PRTEE questionnaire • Grip strength (using a hydraulic hand dynamometer)	• CS injection improved all outcomes at a faster rate over the first 15 days (*p* = 0.0001), and then started to decline slightly until day 90. • After AB injection, all three scores improved steadily and were eventually better (*p* = 0.0001). • 38 (95%) of participants with AB injection and 25 (62.5%) of participants with CS injection achieved complete recovery.
Lebiedzinskieet al. (2015) PEDro = 6,	*n* = 99 participants; ACP group: 53 Mean Age: 47.0 yrs. 28 males, 25 females CS: 46 Mean Age: 54.0 yrs. 12 males, 34 females	• Participants received one injection in the ECRB of ACP or CS (one ml betamethasone injections and two ml of 1% lignocaine) • Baseline evaluation of DASH and re-evaluation at 6 weeks, 6 months, and 1 year.	• DASH: 15-point increase set as significant change	• The steroid group had better mean DASH scores at 6 weeks (*p* = .01) and tended to have lower scores at 6 months (*p* = .05). • At 1 year, the mean DASH score was significantly better in the ACP group (*p* < .05).
Wolf et al. (2011), PEDro = 6	*n* = 28 participants; Saline group: 10 AB group: 9 CS group: 9 Overall average age: 49 yrs. 16 males, 12 females	• Participants received each injection with one mL lidocaine mixed with two mL of designated injection placed under the extensor origin. • Every provided with a standard sheet of stretching exercises. • Outcomes assessed at baseline, and at 2 weeks, 2 months and 6 months after injection.	• DASH • VAS • PRTEE	• No significant between group differences in DASH scores were found at the two- and 6-months assessments. • There were significant improvements in all three groups (*p* < 0.001) over the course of 6 months for the DASH.

*PEDro* Physiotherapy Evidence Database Research Organisation, *AB* Autologous blood, *CS* Corticosteroid, *ECRB* Extensor Carpi Radialis Brevis, *VAS* Visual Analog Scale, *DASH* Disabilities of the Arm, Shoulder and Hand, *PRTEE* Patient Rated Tennis Elbow Evaluation, *qDASH* Quick form of DASH

**Table 2:** Details of studies comparing PRP and corticosteroid injections

Reference/ PEDro score Sackett’s Evidence Level	Participants	Methods	Outcome Measures	Results
Gosens et al. (2011), PEDro = 9	*n* = 100 participants PRP group:51, Age: 46.8 ± 8.5 yrs. 23 males, 28 females CS group: 49 Age: 47.3 ± 7.8 yrs. 23 males, 26 females	• Participants received CS injection (one mL with bupivacaine hydrochloride 0.5%) or PRP injection (three mL buffered with 8.4% sodium bicarbonate and bupivacaine hydrochloride 0.5%) • Injections provided in common extensor tendon through a peppering needling technique. • Outcomes assessed at baseline and at 1 month, 2 months, 3 months, 6 months, 1 year and 2 years after the injection.	• VAS • DASH	• CS group had lower pain and improved on DASH scores at 1 month. • No between group changes at 2 months • PRP group had better outcomes at all other assessments.
Gautam et al. (2015) PEDro = 6	*n* = 30 participants PRP group = 15 CS group = 15 No information provided about age or sex distribution	• Injection delivered using peppering technique at most tender point over lateral epicondyle of humerus • Participants received two ml of PRP or 40 mg/ml of methylprednisolone. • Outcomes assessed before and at 2 weeks, 6 weeks, 3 months, and 6 months after injection. • USG was performed before and after injection at three and 6 months	• VAS, • DASH • Oxford Elbow score • Modified Mayo Clinic performance index for elbow, • Hand grip strength	• All outcome measures improved significantly from pre-injection to 6-month retention in both groups. • CS group had greater changes at two- and 6-weeks post-injection. However, the scores of CS group peaked at 3 months and deteriorated at 6 months • No between groups differences present at 3 months • In the CS group, patients with reduced thickness of tendon increased from two to 12. • PRP group had better within group changes in outcomes at 6 months (*p* < 0.05).
Yadav et al. (2015) PEDro = 5	*n* = 60 participants PRP group: 30 Mean Age: 36.6 yrs. 10 males, 20 females CS group: 30 Mean age: 36.6 yrs. 7 males, 23 females	• Both groups received injection into common extensor origin. • PRP: single injection (one ml), with absolute platelet count of 1 million platelets/ mm^3^ • CS: single injection of corticosteroid (methylprednisolone, 40 mg in one ml) • Data collected at baseline and 15 days, one and 3 months after injection.	• VAS, • grip strength • qDASH	• CS group had statistically significant and better improvement than PRP group at 15 days and at the 1 month follow assessment. • At end of 3 months, VAS, qDASH and grip strength was significantly better in PRP group (*p* < .0001).
Krogh et al. (2013), PEDro = 8	*n* = 60 participants: 20 saline, Age: 44.7 ± 7.9 yrs. 9 males, 11 females 20 PRP, Age: 47.6 ± 7.1 yrs. 9 males, 11 females 20 CS Age: 45.4 ± 8.0 yrs. 11 males, 9 females	• Injections provided using an ultrasound-guided, antiseptic peppering technique in the common extensor origin. • The three ml consisted of one ml of triamcinolone (40 mg/ml) and two ml of lidocaine (CS group), three ml of saline or three ml of PRP. • All participants prescribed a standard stretching and training program. • Outcomes assessed at baseline, and at one and 3 months after injection.	• PRTEE • USG changes in tendon thickness • Color Doppler activity	• CS group had maximum reduction in pain and DASH scores at 1 month. Assessment. • All groups improved at 3 months assessment period with no between group differences. • Maximum reduction in tendon thickness and color doppler outcomes in the CS group at 3 months PRP group had greater changes in tendon thickness and Doppler outcomes compared to saline.

*PEDro* Physiotherapy Evidence Database Research Organisation, *PRP* Platelet rich plasma, *CS* Corticosteroid, *ECRB* Extensor Carpi Radialis Brevis, *VAS* Visual Analog Scale, *DASH* Disabilities of the Arm, Shoulder and Hand, *PRTEE* Patient Rated Tennis Elbow Evaluation, *qDASH* Quick form of DASH

Most commonly used outcomes across all studies included the Visual Analog Scale (VAS) for assessing pain and the Disabilities of the Arm, Shoulder and Hand (DASH) and quick DASH outcomes assessing self-perceived upper limb use in daily life activities. As these were the most-commonly reported outcomes, we performed our meta-analyses using these two outcomes.

### Effects of the provision of regenerative injections on pain levels

**Fig. 3:**
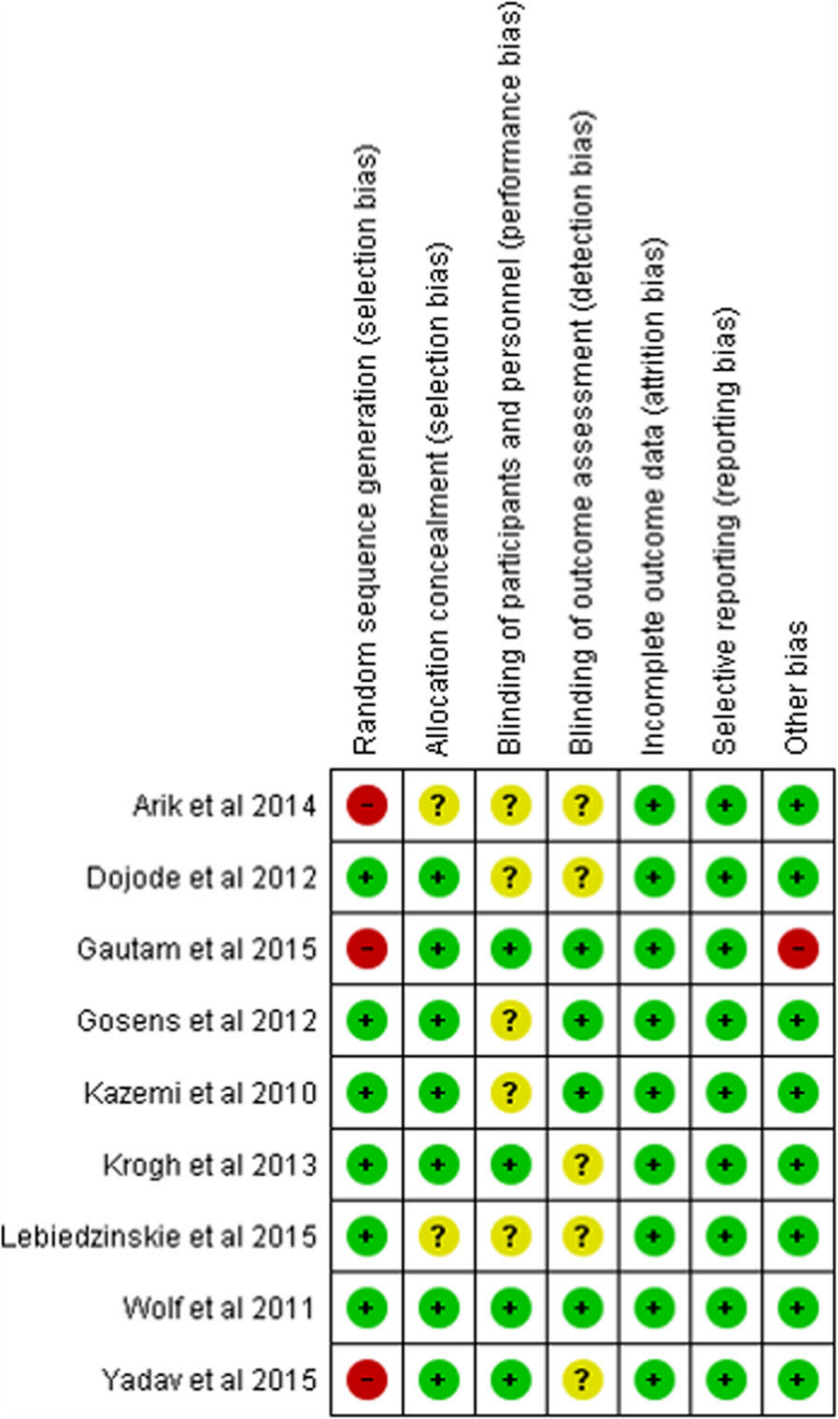
Results of meta-analyses examining the effectiveness of the corticosteroid injections compared to regenerative injections on pain using the VAS scale at 1 month (**a**), 2 months (**b**), 3 months (**c**) and 6 months (**d**) post-injection. Larger squares indicate bigger study effect sizes. The diamonds represent pooled effects of results of individual studies. The location of the diamond indicates the estimated effect size and precision of the estimate is indicated by the width of the diamond

The VAS was used to assess pain levels after the provision of corticosteroid or regenerative injections at four different time points: 1 month [36–38, 42, 44], 2 months [37, 40, 41], 3 months [38, 41, 42, 44] and 6 months post-injection [36, 38, 40, 41, 43]. There were no differences in the amount of pain reported after 1 month (Hedges g: 0.35, 95% CI: − 0.27 to 0.96, small effect size; *p* = 0.27; Fig. 3a) and at 2 months (Hedges g: -0.25, 95% CI: − 1.27 to 0.76, small effect size; *p* = 0.62; Fig. 3b). A significant reduction in pain levels with provision of regenerative injections was noted at three (Hedges g: -0.36, 95% CI: − 0.59 to − 0.12, small effect size; *p* = 0.003; Fig. 3c) and 6 months (Hedges g: -0.73, 95% CI: − 1.14 to − 0.33, moderate effect size; *p* < 0.001; Fig. 3d).

### Effects of the provision of regenerative injections on self-perceived upper limb use in daily life activities

**Fig. 4:**
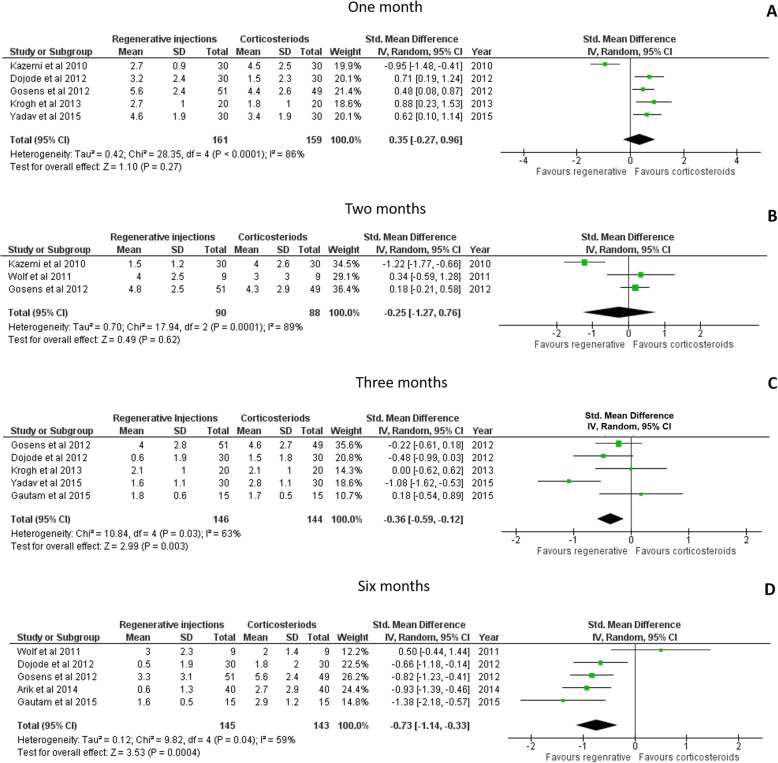
Results of meta-analyses examining the effectiveness of the corticosteroid injections compared to regenerative injections on self reported upper limb use in daily life activities using the DASH scale at 3 months (**a**), 6 months (**b**) and one-year (**c**) post-injection. Larger squares indicate bigger study effect sizes. The diamonds represent pooled effects of results of individual studies. The location of the diamond indicates the estimated effect size and precision of the estimate is indicated by the width of the diamond

The DASH was used to assess self-perceived upper limb use after the provision of corticosteroid or regenerative injections at three different time points: 3 months [36, 39, 40] and 6 months [36, 39–41] and at one-year post-injection [36, 39]. Individuals receiving regenerative injections reported a significant improvement (with small effect sizes) in upper limb use at 3 months (Hedges g: -0.36, 95% CI: − 0.69 to − 0.04, *p* = 0.03; Fig. 4a). Individuals continued to improve at 6 months (Hedges g: -0.32, 95% CI: − 0.58 to − 0.06, *p* = 0.02, Fig. 4b) and at 1 year (Hedges g: -0.45, 95% CI: − 0.73 to − 0.17, *p* = 0.002; Fig. 4c).

## Discussion

We found evidence supporting the provision of regenerative injection therapy as a conservation treatment in individuals with lateral epicondylitis. Individuals receiving these injections had long- term pain relief and increased self-perceived upper limb use. The results also suggest that injections of whole autologous blood as well as platelet rich plasma are useful to decrease pain and increase self-rated upper limb use in individuals with lateral epicondylitis. An additionally important consideration is that the regenerative injections were useful in the acute as well as chronic stages of lateral epicondylitis.

### Use of subjective assessments

The outcomes used in this study assessed the body structure and body function as well as activities of daily living domains of the ICF. When analyzing the literature regarding chronic elbow tendinopathies, we found that treatment effectiveness was quantified using a variety of outcomes. There was a preponderance of the use of subjective outcome measures that included the VAS, Patient Rated Tennis Elbow Evaluation (PRTEE), Oxford Elbow Score and Nirschl score for pain and DASH and quick DASH for arm function. Only one study assessed pain in grip formation and pain pressure threshold, a more objective assessment of pain. Thus, it is currently unclear as to which assessment is most appropriate or whether it is better to use more than one outcome**.** It might be a better idea to use an objective as well as subjective assessment of pain, as the use of a single outcome may not present the entire picture and the use of more than one measure is sometimes warranted [45].

### Use of objective assessments

Objective assessments of body function included the grip strength assessments employed in four studies and ultrasound guided (USG) assessments in two studies. Specifically, these two studies assessed the effects of platelet rich plasma injections. Provision of platelet rich plasma injections resulted in better tendon thickness outcomes compared to corticosteroid at 3 months and 6 months after the injection (Table 2)**.** While the first study [41] did not present specific values in terms of tendon thickness, only one out of 15 participants had reduced thickness of the common extensor tendon. These participants in addition had greater functional improvements. The mean reduction in tendon thickness in the second study ranged from 0.5 to 0.8 mm [42], which is identical to the range of smallest detectable change in healthy individuals [46]. USG findings are reliable for measurement of tendon thickness at the elbow in individuals with lateral epicondylitis [47]. It is currently unknown whether the range of smallest detectable change is similar in individuals with lateral epicondlylitis.

In addition, no study included in the current review evaluated the effects of the provision of autologous blood injections results on changes in tendon morphology as measured by USG and color Doppler outcomes**.** However, other studies have reported a reduction in tendon thickness and hyperechoic changes on USG, with the use of autologous blood injections [48]. Thus, the use of USG to measure tendon thickness may be encouraged as a more objective measurement to understand the effects of regenerative injections on body structures, in addition to body function and activity levels of the ICF. In addition, the findings support the use of grip strength as an objective functional outcome in this population.

### Follow-up assessments

The period for which the treatment provides symptom relief accompanied by functional improvement is an important factor to consider when determining a specific treatment’s efficacy. Four fair to good quality studies included in this review investigated short-term effects of corticosteroid and regenerative injections for lateral epicondylitis with follow-up assessments until 2–3 months post-injection. Individually, in the systematic review, results of three articles [37, 43, 44] reported significant short-term improvements at 1 month for all outcomes in the corticosteroid group (Tables 1 and 2). The relatively higher risk of bias (Fig. 2) in these three studies do limit the generalizability of their findings. In addition, when included in the meta-analysis, no differences between groups were present in terms of pain, as measured using the VAS (using the random effects model due to high heterogeneity, Fig. 3a). Besides, in these three studies, at the 3 months follow-up assessment, the improvements due to corticosteroid had declined and the group receiving regenerative injections continued to improve with respect to pain and self-perceived upper limb use (Figs. 3c and 4a).

Five studies evaluated the effects of injections on pain at a six-month follow up and four studies examined the effects of the injections on self-perceived arm use (Figs. 3d and 4b). In these studies, the patients in the platelet rich plasma group continued to improve and had significantly better results concerning decreased level of pain, improvement of function, and evidence of tendon healing at the six-month follow up. Results of one article [40] comparing corticosteroid, autologous blood, and saline injections demonstrated no significant differences between groups with all participants improving significantly at the six-month follow up. Lack of details regarding exact time from initial diagnosis and the type of corticosteroid injected confound the interpretation of these results and probable reasons to explain the lack of between group changes. However, the results of our meta-analysis demonstrate significant improvement in pain and function at 6 months post-injection in the group receiving regenerative injections.

Lastly, two studies evaluated the effects at one-year post-injection and one study had an assessment at 2 years. At the one-year follow-up assessments, greater improvements in pain relief and functional performance were present in the groups receiving RIT injections. In the study by Gosens et al. [36], at the two-year post-injection assessment, the corticosteroid group had returned to their baseline DASH scores and had mild improvements of pain while the autologous blood group had sustained the level of recovery with significant improvements in the VAS and DASH scores.

When considering the variable of time with respect to the efficacy of corticosteroid compared to regenerative injections, it is evident that in the short-term (one to 2 months), our results indicate that use of corticosteroid injections is not superior to regenerative injections. These findings are similar to results obtained by Sirico and colleagues, who found no short-term benefit for corticosteroid presentation [9]. Our results also agree with those found from other metanalyses in this area of study [49–51] in terms of lack of efficacy of corticosteroids injections in the longer term. However, ours is the only study that considered the effects of provision of both autologous blood as well as platelet rich plasma compared to corticosteroid injections. Additionally, we looked at both pain and function, something that was not a primary focus in the other meta-analyses. Provision of regenerative injections seem to result in long-term improvements lasting until one to 2 years.

### Platelet rich plasma versus autologous blood injections

Amongst the trials included in this study, five and four articles investigated the effects of provision of autologous blood and platelet rich plasma injection provision respectively to corticosteroid injections. As mentioned previously, platelet rich plasma injections are a variation of the autologous blood injection technique, where there is a greater amount of whole blood removed from the patient and centrifuged to produce a higher concentration of platelets for healing [12, 15]. For both platelet rich plasma and autologous blood injection studies, seven of the studies found significant, beneficial effects of the regenerative injections while two found the injections were not significantly superior to placebo saline injections. A systematic review by Thøger Persson et al. [52] found similar results comparing many types of injections for effectiveness in lateral epicondylitis, including two trials for platelet rich plasma and three for autologous blood. A recent network meta-analysis [53] found no difference between autologous blood and platelet rich plasma injections. Thus, it seems that regenerative injections are useful for the long-term resolution of symptoms and the kind of injection does not matter.

### PEDro scale and risk of bias

As stated earlier, the quality of the studies based on PEDro scales ranges from excellent (one study), good (six studies) to fair (two studies). None of the studies were rated as being of poor quality. The two studies ranked as fair [43, 44] also had a high risk of selection bias (on random allocation) in addition to unclear risk on detection bias (Fig. 2). While the items on the PEDro scale talk about randomization and concealed allocation, they do not need specific information on how the process of randomization and allocation were carried out. There are a few guidelines available like the non-acceptability of coin-toss methods. However, this information may be insufficient.

The unclear risk of detection bias in other studies was due to the inadequate information on whether blinded assessors were employed and some of them used objective outcomes including grip force measurement using dynamometry. Inadequate information on blinded assessors can lead to a risk of assigning scores to the intervention group indicative of better recovery. One study [41] had a high risk of other bias in terms on non-provision of information regarding average age and sex distribution of participants. This study was of good quality, scoring 6/10 in the PEDro scale. The PEDro scale, however, does not account for sample size and age and sex distribution. This study did not have information on random allocation and scored lower in that regard on the PEDro. Future studies must ensure inclusion of all relevant details to avoid the risk of bias and help ensure correct reporting standards by using both the PEDro scale and Risk of Bias tool to help better interpret their results.

### Heterogeneity in results of the metanalyses

The results of our metanalyses for pain and self-perceived function revealed considerable heterogeneity. Factors that could have contributed to the heterogeneity include a wide range of sample sizes across all studies (ranging from 18 to 100 participants) and choice of the outcome measures. Only four [36, 37, 40, 42] had explicit sample size analyses mentioned in the paper. In addition, as mentioned above, the PEDro scale does not take sample size into account. Future studies must include an explicit sample size analysis to understand the rationale behind the numbers of participants in the study. In terms of outcome measures, data was available for the VAS and DASH (subjective outcomes) as a measure of pain and functional performance respectively. It remains to be seen if the use of more objective outcomes (without the risk of detection bias) helps reduce heterogeneity amongst studies.

### Limitations and future directions

It is important to acknowledge the limitations and gaps in the literature presented in this review. Out search was limited to papers published in English between June 2008 and May 2018. Although we found no RCTs directly comparing corticosteroids to either autologous blood and/or platelet rich plasma injections published in English prior to 2008 in our literature search, we may have missed some articles published in other languages. This issue may have introduced a possible selection bias. A lack of standardization for the preparation of regenerative injections was present amongst the different studies. There was also no control regarding prior use of either injection or receiving multiple injections during the study [36, 42]. There was a high risk of selection bias in three studies and unclear risk of performance and detection bias in other studies that could have influenced the results. The use of a meta-analytic approach helps safeguard against some of these risks, as it pools all the data together. However, these results need to be interpreted with due consideration to the different types of bias. In addition, only three out of the nine studies [36, 40, 42] provided details regarding prescription of exercises after the provision of RIT or corticosteroid injections. Future studies should investigate the effects of: i) a single corticosteroid or regenerative injection when compared to multiple injections for the treatment of lateral epicondylitis; ii) a standardized exercise protocol provided after the injections on levels of pain and self-rated disability between groups; iii) autologous blood injections on tendon thickness assesses using USG and color Doppler outcomes; and iv) development of a standardized protocol for preparation of regenerative injections.

## Conclusion

Lateral epicondylitis can be a chronic condition that tends to influence the quality of life of people afflicted by the condition severely. For this reason, individuals with lateral epicondylitis are constantly searching for alternative therapies to expedite healing time. While corticosteroid injections are a common option, the use of regenerative injections has been successful in clinical trials with long-term follow-ups and signs of tendon repair. While both types of injections aim to provide relief, the mechanism of action varies. This in turn leads to differing effects on the tendon and healing process. Our results suggest that regenerative injections are useful in the long-term with no short-term differences in pain-reduction between corticosteroid and regenerative injections.

## Data Availability

Data sharing not applicable to this article as no datasets were generated or analyzed during the current study.
