# Correction to: The effects of regenerative injection therapy compared to corticosteroids for the treatment of lateral Epicondylitis: a systematic review and meta-analysis

**DOI:** 10.1186/s40945-020-0076-1

**Published:** 2020-01-27

**Authors:** Julie Barnett, Madison N. Bernacki, Jessica L. Kainer, Hannah N. Smith, Annette M. Zaharoff, Sandeep K. Subramanian

**Affiliations:** 1 Department of Physical Therapy, School of Health Professions, UT Health San Antonio, 7703 Floyd Curl Drive, MSC 6247, San Antonio, TX 78229 USA; 2 The Non-Surgical Center of Texas, San Antonio, USA

**Correction to: Arch Physiother (2019) 9:12**

**https://doi.org/10.1186/s40945-019-0063-6**

In the original version of this article [1] the legends of Figs. 2 and 3 were inadvertently interchanged.

Figure 2 presents the results of meta-analyses examining the effectiveness of the corticosteroid injections compared to regenerative injections on pain using the VAS scale at 1 month (a), 2 months (b), 3 months (c) and 6 months (d) post-injection.

Figure 3 presents the risk of bias summary for included RCTs.

The figure legends have already been updated in the original article [1].

## References

[1] Barnett (2019). The effects of regenerative injection therapy compared to corticosteroids for the treatment of lateral Epicondylitis: a systematic review and meta-analysis. Arch Physiother.

